# Accuracy of Surveys for Estimating Coverage for Hepatitis A and B Vaccinations in Adults

**DOI:** 10.5888/pcd23.250481

**Published:** 2026-05-21

**Authors:** Lori A. Crane, Laura P. Hurley, Matthew F. Daley, Brenda Beaty, John D. Rice, Kamonthip J. Homdayjanakul, Jason Lyons, Carla L. Black, Peng-Jun Lu, James A. Singleton

**Affiliations:** 1 Department of Community and Behavioral Health, Colorado School of Public Health, University of Colorado Anschutz, Aurora, Colorado; 2 Division of General Internal Medicine, Denver Health, Denver, Colorado; 3 Department of Medicine, University of Colorado Anschutz, Aurora, Colorado; 4 Institute for Health Research, Kaiser Permanente Colorado, Aurora, Colorado; 5 Department of Pediatrics, University of Colorado Anschutz, Aurora, Colorado; 6 Adult and Child Center for Outcomes Research and Delivery Science, University of Colorado Anschutz, Aurora, Colorado; 7 Department of Biostatistics, University of Michigan School of Public Health, Ann Arbor, Michigan; 8 Immunization Services Division, National Center for Immunization and Respiratory Diseases, Centers for Disease Control and Prevention, Atlanta, Georgia

## Abstract

This study estimated accuracy of survey-reported hepatitis A and B vaccination among adults at increased risk. Survey responses from patients at 2 large health care systems in Colorado were compared with vaccination records in electronic health records and the state immunization registry. For hepatitis A vaccine, net bias was 5.6% (relative bias, 38.6%; sensitivity, 52.5%; specificity, 85.3%). For hepatitis B vaccine, net bias was 6.5% (relative bias, 28.6%; sensitivity, 43.4%; specificity, 75.1%). Despite low sensitivity, self-reported vaccination status may be sufficiently accurate for use in the development of vaccine policies and public health actions for improving hepatitis A and B coverage.

**Summary**

**What is already known on this topic?**

Surveys are commonly used for surveillance of health behaviors, including receipt of vaccinations. However, little is known about the accuracy of survey-based self-reports of vaccination status.

**What is added by this report?**

In this study of patients considered at elevated risk for hepatitis A, hepatitis B, or both, we found moderate over-reporting of vaccination for hepatitis A and hepatitis B when self-report was compared with vaccination records in the electronic health record and the Colorado Immunization Information System.

**What are the implications for public health practice?**

Accuracy of self-report was in an acceptable range for use in development of vaccine policies and public health actions for improving hepatitis A and B vaccination coverage.

## Objective

Hepatitis A (HepA) and B (HepB) are vaccine-preventable infections. HepA is usually acute and can be severe. HepB can be chronic, increasing the risk of liver failure and cancer. HepA is usually transmitted through the fecal–oral route or through contaminated food or water; HepA vaccination recommendations for adults are risk-based, focusing on sexual contact, drug use, occupational risk, homelessness, HIV, and chronic liver disease (1). HepB is transmitted through blood. From 1982 until 2022, HepB vaccination was recommended for high-risk adults, which included those with drug use and multiple sexual partners. Starting in 2022, HepB vaccination was expanded as a universal vaccination recommendation for adults through age 59 years (2).

Accurate data are needed to monitor vaccination coverage. In the US, no single comprehensive source of vaccination information exists. Large national surveys are used, but little is known about the accuracy of survey-based self-reports of vaccination status. The National Health Interview Survey (NHIS) has been used to estimate hepatitis vaccination coverage (3,4). Most recently, in 2021, self-reported hepatitis vaccination coverage ranged from 25% to 40%, with higher coverage among those with risk factors and higher coverage for HepB compared with HepA (4).

Our aim was to estimate the sensitivity, specificity, net bias, and relative net bias of self-reported hepatitis vaccination status among adults at increased risk for HepA, HepB, or both. This study can be situated within the Total Survey Error (TSE) framework, which conceptualizes error in survey estimates as arising from multiple sources. Our study focused on measurement error (5).

## Methods

This study was conducted in Denver Health, the public safety-net health system for the city and county of Denver, Colorado, that serves 177,000 patients annually, and Kaiser Permanente Colorado, a membership-based, integrated health system that serves more than 500,000 members in the front-range urban corridor where more two-thirds of Colorado’s population resides.

Using electronic health records (EHRs) and International Classification of Diseases (ICD) 9 and ICD 10 codes, we identified current patients aged 30 years or older with risk factors for HepA (eg, illicit drug use), HepB (eg, history of sexually transmitted diseases), or both (eg, chronic liver disease) (Appendix). People were considered to be vaccinated for HepA if the EHR recorded at least 2 doses and for HepB if the EHR recorded 3 or more standard doses or 2 or more adjuvanted doses (6). We randomly sampled equal proportions of vaccinated and unvaccinated people across the 2 systems. Patients were eligible if they spoke English or Spanish, did not have a cognitive impairment or disability that prevented them from responding, and had at least 1 valid form of contact (email, postal address, or telephone).

The survey, introduced as a survey about community health (Table 1), was available in English and Spanish. Questions were drawn from the 2021 NHIS (receipt of vaccine), 2018 NHIS (number of doses), and 2021 Behavioral Risk Factor Surveillance System (BRFSS; demographic questions) (Table 1) (7–9). Survey administration was during January through April 2022. Contacts included a postal letter, up to 3 email reminders to complete the survey online, up to 8 telephone calls to complete the survey by telephone, and a final email reminder. Respondents received a $10 gift card for participating.

**Table 1:** Survey Introduction and Question Script Used to Assess Receipt of Hepatitis A and B Vaccination, Survey of Colorado Adults at Increased Risk for Hepatitis A and B, 2022

Survey component	Wording
Survey introduction in invitation letter (postal mail and email)	Dear [name]: I’m writing to let you know that [Healthcare System] is working with the Colorado School of Public Health at the University of Colorado and the national Centers for Disease Control and Prevention (CDC) in a research study of health and health practices. You have been randomly selected from among our patients at [Healthcare System] to participate in the research study. [Site Principal Investigator] at [Healthcare System] is working with the study team. [Healthcare System] recently sent a letter to you at your home address about this study. Your participation in the research study is important. The findings will be used to improve the health of your community and our nation. It is important for the study to have a high response rate to have valid results. We at [Healthcare System] encourage you to participate. **Please read the following information about this study:** We are asking you to complete a survey that will take only about **7 minutes** of your time. The study will give you a **$10 electronic gift card** if you complete the **survey**. This will be emailed or mailed to you within 2–3 weeks after you complete the survey. You can complete the survey **online** by going to the link at the end of this letter. Or you can complete the survey **over the phone**. To complete the survey over the phone, you can simply wait, and an interviewer will call you. Or, you can call the number at the bottom of this letter. You will be called from the University of Wyoming, who is also a partner in this study. Participating in this research is **completely voluntary**. You do not have to answer any survey question you do not want to answer. You can end the survey at any time. **All the information you give will be kept private**. The results will not be connected to your name and will not be given to your health care provider. **If you have any questions** about the study or your rights as a research participant, you can call the principal investigator of the study at the Colorado School of Public Health, [name and phone number]. Or, you can call the Colorado Multiple Institutional Review Board, which oversees the safety of research studies [phone number]. **If you decide that you do *not* want to participate** and do *not* want to be contacted by phone for this survey, please send an email message to [email address] or leave a voicemail message at [phone number]. Please remember to include your name, the access code below in this letter, and the words “opt-out.” By agreeing to participate in the survey, you are agreeing to be a part of this research. The data we collect will be used for this study and may also be used for future research. If it is used for future research, all information that could identify you will be removed. I hope you will consider completing the survey. This will help improve the health of our community and nation.
Survey description in introduction to survey (telephone version)	We are offering a $10 gift card for a short 7–10 minute survey about the health and health practices of Colorado residents. The survey is part of a research project conducted by the Colorado School of Public Health, your health care provider [Healthcare System] and the Centers for Disease Control and Prevention, also known as the CDC. [Healthcare System] recently sent you a letter to let you know that you have been randomly selected to participate in this research and that we would be calling. This call may be recorded for quality purposes. May I continue? Your participation in this survey is important because it serves as a way to improve the health of your community and our nation. If you choose to participate, your confidential responses will help provide meaningful information about public health topics.
Survey description in introduction to survey (internet version)	Thank you for logging into this survey about health and health practices in Colorado. If you complete the survey, you will be sent a $10 electronic gift card. This will be emailed to you within 2–3 weeks after you complete the survey. This survey is part of a research project being conducted by the Colorado School of Public Health, in collaboration with [Healthcare System] and the Centers for Disease Control and Prevention (CDC). Your participation in this survey is important because it serves as a way to improve the health of your community and our nation. If you choose to participate, your confidential responses will help provide meaningful information about public health topics.
Assessment of hepatitis A vaccination (source: NHIS 2021)	The hepatitis A vaccine is given in two separate doses. Although it can be given as a combination vaccine with hepatitis B, it is different from the hepatitis B vaccine. Have you EVER received the hepatitis A vaccine? *Read if necessary for telephone survey; provided to all for internet survey:* The hepatitis A vaccine has been available since 1995. It is routinely given to some children starting at 1 year of age and to some adults and people who travel outside the United States.
Number of hepatitis A shots received (source: NHIS 2018)	How many hepatitis A shots did you receive?
Assessment of hepatitis B vaccination (source: NHIS 2021)	The hepatitis B vaccine is given in three separate doses. Although it can be given as a combination vaccination with hepatitis A, it is different from the hepatitis A vaccine. Have you EVER received the hepatitis B vaccine? *Read if necessary for telephone survey; provided to all for internet survey:* The hepatitis B vaccine has been available since 1991. It is recommended for newborn infants, adolescents, and people such as health care workers, who may be exposed to the hepatitis B virus.
Number of hepatitis B shots received (source: NHIS 2018)	Did you receive at least 3 doses of the hepatitis B vaccine or less than 3 doses?

Abbreviation: NHIS, National Health Interview Survey.

Unequal sampling probabilities were accounted for by using sampling weights based on each stratum’s eligible population and number of respondents. We used SAS PROC SURVEYFREQ to assess sensitivity, specificity, net bias, and relative net bias (SAS Institute, Inc) (10). Sensitivity and specificity were calculated at the individual level by comparing each respondent’s self-reported vaccination status with the criterion standard. The criterion standard included vaccination information from the EHR, supplemented with records from the Colorado Immunization Information System (CIIS), which compiles data on vaccines received throughout Colorado. Net bias is a population-level summary, calculated as the self-reported vaccination coverage at the population level minus the criterion standard vaccination coverage. Relative net bias is net bias divided by the criterion standard vaccination, multiplied by 100. Survey responses of “don’t know” for vaccination status or number of doses received were excluded (complete cases approach), which is consistent with analysis methods used in published reports of self-reported vaccination status in national surveys. This approach treats uncertain responses as missing and therefore does not distinguish between misreporting and uncertainty.

The Colorado Multiple Institutional Review Board approved the study.

## Results

After removing 3 ineligible patients, the HepA sample included 492 adults; after removing 8 ineligible patients, the HepB sample included 510 adults. Response rates were 38.2% (HepA) and 40.0% (HepB). For HepA, response status was significantly related to age and health care system (Table 2). For HepB, response status was related to age, race and ethnicity, study site, and insurance type. Response status was not significantly related to vaccination status for HepA or HepB.

**Table 2:** Characteristics of Respondents and Comparison to Nonrespondents, Survey of Colorado Adults at Increased Risk for Hepatitis A and B, 2022

	Hepatitis A risk group survey response (N = 492)			Hepatitis B risk group survey response (N = 510)		
Variable	No, % (n)	Yes, % (n)	*P* value^a^	No, % (n)	Yes, % (n)	*P* value^a^
**Overall**	61.8 (304)	38.2 (188)	NA	60.0 (306)	40.0 (204)	NA
**Sex**						
Female	43.1 (131)	42.6 (80)	.91	49.3 (151)	47.5 (97)	.69
Male	56.9 (173)	57.4 (108)		50.7 (155)	52.5 (107)	
**Age group, y**						
30–49	38.2 (116)	33.0 (62)	.008	22.9 (70)	20.1 (41)	.02
50–64	37.2 (113)	29.3 (55)		42.8 (131)	33.3 (68)	
≥65	24.7 (75)	37.8 (71)		34.3 (105)	46.6 (95)	
**Race and ethnicity**						
Non-Hispanic Black	12.5 (38)	11.7 (22)	.42	13.1 (40)	10.8 (22)	.02
Non-Hispanic White or Other	60.9 (185)	66.5 (125)		50.3 (154)	62.7 (128)	
Hispanic	26.6 (81)	21.8 (41)		36.6 (112)	26.5 (54)	
**Study site**						
Large, safety-net, urban	50.0 (152)	38.8 (73)	.02	54.2 (166)	34.8 (71)	<.001
Integrated health care organization	50.0 (152)	61.2 (115)		45.8 (140)	65.2 (133)	
**Insurance type**						
Medicare	34.2 (104)	41.0 (77)	.44	40.2 (123)	52.0 (106)	.04
Medicaid	33.9 (103)	28.2 (53)		26.5 (81)	18.1 (37)	
HMO or commercial	27.6 (84)	27.1 (51)		24.5 (75)	23.5 (48)	
Other, unknown, or uninsured **b**	4.3 (13)	3.7 (7)		8.8 (27)	6.4 (13)	
**Vaccinated based on criterion standard^c^**						
Yes	49.7 (151)	56.4 (106)	.15	46.1 (141)	53.9 (110)	.08
No	50.3 (153)	43.6 (82)		53.9 (165)	46.1 (94)	
**Education level**						
High school graduate/GED or less	—d	27.8 (52)	NA	—d	30.0 (61)	NA
Some college or technical school	—d	32.1 (60)		—d	27.6 (56)	
College graduate or more	—d	40.1 (75)		—d	42.4 (86)	
**Annual household income, $^e^**						
<75,000	—d	68.3 (114)	NA	—d	66.9 (121)	NA
≥75,000	—d	31.7 (53)		—d	33.1 (60)	

Abbreviations: GED, general educational diploma; HMO, health maintenance organization; NA, not applicable.

a *P* value based on χ^2^ test.

b Other insurance includes self-pay, Veterans Affairs, Tricare, correctional, and dual-eligible.

c Criterion standard included vaccination information from the electronic health record supplemented with records from the Colorado Immunization Information System, which compiles vaccines received from across Colorado.

d Data collected only for survey respondents.

e For hepatitis A risk group, 21 were missing household income (11%). For hepatitis B risk group, 23 were missing household income (11%).

Vaccination coverage for HepA based on the criterion standard for the respondent population (weighted) was 16.1%, which was one-third higher than the EHR vaccination coverage for the source population (11.7%), in part demonstrating the influence of incorporating vaccination information from the CIIS (Table 3). After removing survey respondents who reported they “don’t know” whether they have been vaccinated for HepA, the criterion standard vaccination coverage was 14.6%. Self-reported vaccination status for these people was higher, at 20.2%, resulting in a net bias of 5.6% (relative net bias 38.6%), which indicates over-reporting of vaccination. Sensitivity was 52.5% and specificity was 85.3%.

**Table 3:** Vaccination Coverage Estimates and Accuracy Statistics for Self-Report of Vaccination Status, Survey of Colorado Adults at Increased Risk for Hepatitis A and B, 2022

Characteristic	Hepatitis A (2 doses)	Hepatitis B (3 doses)
Source population, n^a^	35,262	84,079
EHR vaccination coverage for source population (%)^a^	11.7	11.1
Sampled population, n^a^	492	510
Respondent population, n^a^	188	204
Criterion standard vaccination coverage for respondent population, weighted %^b^	16.1	22.3
Respondents who said they “don’t know” their vaccination status, weighted %	35.4	35.6
For those who said “don’t know,” proportion vaccinated per criterion standard, weighted %	19.0	21.7
Respondent population after removing those who said “don’t know,” n^a^	115	129
Criterion standard vaccination coverage for surveyed population after removing those who said “don’t know,” weighted %^b^	14.6	22.6
Self-reported vaccination after removing those who said “don’t know,” weighted %	20.2	29.1
Sensitivity, weighted % (95% CI)	52.5 (39.4–65.7)	43.4 (28.6–58.2)
Specificity, weighted % (95% CI)	85.3 (75.9–94.8)	75.1 (64.0–86.2)
Net bias estimate, weighted %^c^	5.6	6.5
Relative net bias, weighted %^c^	38.6	28.6

Abbreviation: EHR, electronic health record.

a Data are unweighted.

b The criterion standard included vaccination information from the EHR supplemented with records from the Colorado Immunization Information System, which compiles vaccines received from across Colorado.

c Net bias was calculated as self-reported vaccination coverage minus criterion standard vaccination coverage. Relative net bias was calculated as (net bias/criterion standard vaccination coverage) × 100.

Vaccination coverage for HepB based on the criterion standard for the respondent population (weighted) was 22.3%, which was double the EHR vaccination coverage for the source population (11.1%). After removing survey respondents who reported they “don’t know” whether they have been vaccinated for HepB, the criterion standard vaccination coverage was essentially unchanged at 22.6%. Self-reported vaccination status for these people was higher, at 29.1%, resulting in a net bias of 6.5% (relative net bias 28.6%). Sensitivity was 43.4% and specificity was 75.1%.

## Discussion

In this study conducted in 2 large health care systems, sensitivity of self-reported vaccination status was low and specificity was moderately high for both HepA and HepB vaccination, indicating that self-report was more accurate for identifying unvaccinated people than vaccinated people. Because the source population was largely unvaccinated, specificity had a greater impact than sensitivity on net bias, resulting in vaccination estimates based on self-report being 5 to 7 percentage points higher than records-based estimates. Using CIIS data increased the records-based estimates by 4 to 11 percentage points over EHR alone, showing the importance of access to records outside of the current medical home.

One previous study examined this question in a single closed health system in the eastern US where the population was primarily non-Hispanic White and insured; that study did not supplement EHR data with immunization information systems data (11). That study also found positive net bias, with patient self-report somewhat more accurate for HepA compared with HepB vaccination.

This study has limitations. Vaccinations received at health care facilities that did not report to the CIIS at the time the vaccination was given, and vaccines that were received in another state, were not included, resulting in underreporting of true status in the criterion standard. The generalizability of this study may be limited because it was conducted in a single state. However, we sampled from 2 large health care systems that serve both insured and uninsured patients across a wide range of sociodemographic backgrounds. We sampled only patients who had at least 1 valid form of contact. Thus, our findings apply to adults who are reachable within these health care systems and may not be generalizable to people who are less connected to care. Comparison of respondents with nonrespondents shows some differences in response status by demographic group. Our response rate was similar to that of the BRFSS (43.7%) and reflects the declining survey response rates observed over recent decades (12,13). While working to increase response rates may be necessary, as Olson has pointed out, increasing the response rate through incentives or intensive contact methods could contribute to increased measurement error bias because respondents who are difficult to reach may be less accurate in reporting their vaccination status (5). Our estimates apply to these specific survey questions and this survey context, and accuracy of reporting may differ for other vaccine questions, other populations, or differently framed surveys. Additionally, our complete-case approach excluded respondents who answered “don’t know.” As discussed by Olson, treating uncertain responses as missing can confound uncertainty and item nonresponse with misreporting, and may also mix compositional differences among those with missing or uncertain responses with measurement error among complete cases (5). As a result, our accuracy estimates pertain to respondents providing definitive yes or no answers.

From a clinical perspective, our results suggest that what patients report to their clinicians may not be accurate. One implication is the need to develop better vaccination documentation systems, including across state jurisdictions. In the absence of documentation of vaccination and lack of certainty from the patient, the best clinical decision may be to offer to vaccinate.

In conclusion, although there was positive net bias, both the self-report and records-based coverage estimates for HepA and HepB vaccinations were low (<30%), and the differences between them were small enough that using either data source would lead to similar policy recommendations and strategies for addressing undervaccination. Thus, we conclude that estimates of vaccination based on self-report surveys may be sufficiently accurate for use in the development of public health actions for HepA and HepB vaccinations. Framed within the TSE perspective, although self-report of vaccination in surveys may be relatively unbiased, survey data may be affected by other biases that were not addressed by our study, including incomplete sample frame coverage and nonresponse. Although survey data may be adequate for estimating population coverage, individual-level analyses could be biased because of misclassification of vaccination status. Due to the lack of a centralized source of vaccination records in the US, surveys remain a necessary and valuable mechanism for population-level vaccination surveillance (14).

## Appendix ICD 9/ICD 10 Codes Used to Sample Adults With Increased Risk for Hepatitis A and B, Colorado, 2022

**Table:**

Condition	ICD 9 code	ICD 10 code
**At risk for hepatitis A and hepatitis B**		
**Chronic liver disease**		
Chronic liver disease and cirrhosis	571	
Alcoholic fatty liver	571.0	K70.0
Alcoholic hepatitis		K70.1
Alcoholic hepatitis without ascites		K70.10
Alcoholic hepatitis with ascites		K70.11
Alcoholic fibrosis and sclerosis of liver		K70.2
Alcoholic cirrhosis of the liver	571.2	K70.3
Alcoholic cirrhosis of liver without ascites		K70.30
Alcoholic cirrhosis of liver with ascites		K70.31
Alcoholic hepatic failure		K70.4
Alcoholic hepatic failure without coma		K70.40
Alcoholic hepatic failure with coma		K70.41
Alcoholic liver disease, unspecified		K70.9
Toxic liver disease		K71
Toxic liver disease with cholestasis		K71.0
Toxic liver disease with hepatic necrosis		K71.1
Toxic liver disease with hepatic necrosis, without coma		K71.10
Toxic liver disease with hepatic necrosis, with coma		K71.11
Toxic liver disease with chronic persistent hepatitis		K71.3
Toxic liver disease with chronic lobular hepatitis		K71.4
Toxic liver disease with chronic active hepatitis		K71.5
Toxic liver disease with chronic active hepatitis without ascites		K71.50
Toxic liver disease with chronic hepatitis with ascites		K71.51
Toxic liver disease with hepatitis, not elsewhere specified		K71.6
Toxic liver disease with fibrosis and cirrhosis of liver		K71.7
Toxic liver disease with other disorders of the liver		K71.8
Toxic liver disease, unspecified		K71.9
Hepatic failure, not elsewhere classified		K72
Chronic hepatic failure		K72.0
Chronic hepatic failure without coma		K72.10
Chronic hepatic failure with coma		K72.11
Hepatic failure, unspecified		K72.9
Hepatic failure, unspecified without coma		K72.90
Hepatic failure, unspecified with coma		K72.91
Chronic hepatitis, not elsewhere classified		K73
Chronic persistent hepatitis, not elsewhere classified		K73.0
Chronic lobular hepatitis, not elsewhere classified		K73.1
Chronic active hepatitis, not elsewhere classified		K73.2
Other chronic hepatitis, not elsewhere classified		K73.8
Alcoholic liver damage, unspecified	571.3	
Chronic hepatitis, unspecified	571.40	K73.9
Fibrosis and cirrhosis of liver		K74
Other and unspecified cirrhosis of liver		K74.6
Unspecified cirrhosis of liver		K74.60
Other cirrhosis of liver		K74.69
Hepatic fibrosis		K74.0
Hepatic sclerosis		K74.1
Hepatic fibrosis and hepatic sclerosis		K74.2
Chronic persistent hepatitis	571.41	
Autoimmune hepatitis	571.42	K75.4
Other	571.49	
Cirrhosis of the liver without mention of alcohol	571.5	
Biliary cirrhosis	571.6	
Primary biliary cirrhosis		K74.3
Secondary biliary cirrhosis		K74.4
Biliary cirrhosis, unspecified		K74.5
Nonspecific reactive hepatitis		K75.2
Granulomatous hepatitis, not elsewhere classified		K75.3
Other chronic nonalcoholic liver disease	571.8	
Unspecified chronic liver disease without mention of alcohol	571.9	
Hepatic coma	572.2	
Portal hypertension	572.3	K76.6
Hepatorenal syndrome	572.4	K76.7
Other sequelae of chronic liver disease	572.8	
Other disorders of the liver	573	
Chronic passive congestion of the liver	573.0	K76.1
Other specified inflammatory liver diseases		K75.8
Nonalcoholic steatohepatitis		K75.81
Other specified inflammatory liver diseases		K75.89
Other diseases of the liver		K76
Fatty(change of liver), not elsewhere classified		K76.8
Central hemorrhagic necrosis of liver		K76.2
Infarction of liver		K76.3
Peliosis hepatitis		K76.4
Hepatic veno-occlusive disease		K76.5
Viral hepatitis B with hepatic coma	070.2	
Viral hepatitis B without mention of hepatic coma	070.3	
Other specified viral hepatitis with hepatic coma	070.4	
Acute or unspecified hepatitis C with hepatic coma	070.41	
Hepatitis delta without mention of active hepatitis B disease with hepatic coma	070.42	
Hepatitis E with hepatic coma	070.43	
Chronic hepatitis C with hepatic coma	070.44	
Other specified viral hepatitis with hepatic coma	070.49	
Other specified viral hepatitis without mention of hepatic coma	070.5	
Acute or unspecified hepatitis C without mention of hepatic coma	070.51	
Hepatitis delta without mention of active hepatitis B disease or hepatic coma	070.52	
Hepatitis E without mention of hepatic coma	070.53	
Chronic hepatitis C without mention of hepatic coma	070.54	
Other specified viral hepatitis without mention of hepatic coma	070.59	
Unspecified viral hepatitis with hepatic coma	070.6	
Unspecified viral hepatitis without mention of hepatic coma	070.9	
Chronic viral hepatitis		B18
Chronic viral hepatitis B with delta agent		B18.0
Chronic viral hepatitis B without delta agent		B18.1
Chronic viral hepatitis C		B18.2
Other chronic viral hepatitis		B18.8
Chronic viral hepatitis, unspecified		B18.9
Unspecified viral hepatitis		B19
Unspecified viral hepatitis with hepatic coma		B19.0
Unspecified viral hepatitis B		B19.1
Unspecified viral hepatitis B without hepatic coma		B19.10
Unspecified viral hepatitis B with hepatic coma		B19.11
Unspecified viral hepatitis C		B19.2
Unspecified viral hepatitis C without hepatic coma		B19.20
Unspecified viral hepatitis C with hepatic coma		B19.21
Unspecified viral hepatitis without hepatic coma		B19.9
**HIV**	042	B20
**High-risk homosexual behavior**		Z72.52
**At risk specifically for hepatitis A** ^a^		
**Noninjection drug use**		
Drug dependence	304	
Opioid type dependence	304.0	
Opioid-related disorders		F11 and F11.X and F11.XX and F11.XXX
Barbiturate and similarly active sedative or hypnotic dependence	304.1	
Sedative, hypnotic, or anxiolytic-related disorders		F13 and F13.X and F13.XX and F13.XXX
Cocaine dependence	304.2	
Cocaine-related disorders		F14 and F14.X and F14.XX and F14.XXX
Cannabis dependence	304.3	
Cannabis-related disorders		F12 and F12.X and F12.XX and F12.XXX
Amphetamine and other psychostimulant dependence	304.4	
Other stimulant-related disorders		F15 and F15.X and F15.XX and F15.XXX
Other specified drug dependence	304.6	
Combination of opioid type drug with any other	304.7	
Combinations of drug dependence excluding opioid type drug	304.8	
Unspecified drug dependence	304.9	
Nondependent drugs of abuse	305	
Cannabis abuse	305.2	
Hallucinogen abuse	305.3	
Hallucinogen-related disorders		F16 and F16.X and F16.XX and F16.XXX
Barbiturate and similarly acting sedative and hypnotic abuse	305.4	
Opioid abuse	305.5	
Cocaine abuse	305.6	
Amphetamine or-related acting sympathomimetic abuse	305.7	
Other, mixed, or unspecified drug abuse	305.9	
Inhalant-related disorders		F18 and F18.X and F18.XX and F18.XXX
Other psychoactive substance-related disorders		F19 and F19.X and F19.XX and F19.XXX
**Homelessness**	V60.0	Z59.0
**At risk specifically for hepatitis B^b^**		
**Sexual exposure risk (includes people seeking evaluation or treatment for a sexually transmitted infection)**		
Early syphilis, symptomatic	091 and 091.X	
Early syphilis, latent	092 or 092.X	
Cardiovascular syphilis	093 or 093.X or 093.XX	
Neurosyphilis	094 or 094.X or 094.XX	
Other forms of late syphilis, with symptoms	095 and 095.X	
Late syphilis, latent	096	
Other and unspecified syphilis	097 and 097.X	
Gonococcal infections	098 and 098.X and 098.XX	A54 and A54.X and A54.XX
Other venereal diseases	099 and 099.X and 099.XX	
Genital herpes	054.1	
Genital warts	078.11	
Unspecified viral and chlamydial infection	079.8	
Other specified chlamydial infection	079.88	
Unspecified chlamydial infection	079.98	
Trichomoniasis	131 and 131.X and 131.XX	A59 and A59.X and A59.XX
Early syphilis		A51 and A51.X
Late syphilis		A52 and A52.X
Other and unspecified syphilis		A53 and A53.X
Chlamydial lymphogranuloma (venereum)		A55
Other sexually transmitted chlamydial diseases		A56 and A56.X and A56.XX
Chancroid		A57
Granuloma inguinale		A58
Anogenital herpesviral [herpes simplex] infections		A60 and A60.X and A60.XX
Other predominantly sexually transmitted diseases, not elsewhere classified		A63 and A63.X
Unspecified sexually transmitted disease		A64
Mycoplasma genitalum		A49.3
Percutaneous or mucosal risk for exposure to blood^c^		
Renal dialysis status	V45.11	
Chronic kidney disease stage 4		N18.4
Chronic kidney disease stage 5		N18.5
End-stage renal disease		N18.6
Dependence on renal dialysis (hemodialysis or peritoneal dialysis)		Z99.2
Diabetes mellitus due to underlying condition (for people aged <60 y)		E08 and E08.X and E08.XX and E08.XXX
Drug or chemical induced diabetes mellitus (for people aged <60 y)		E09 and E09.X and E09.XX and E09.XXX
Type 1 diabetes mellitus (for people aged <60 y)		E10 and E10.X and E10.XX and E10.XXX
Type 2 diabetes mellitus (for people aged <60 y)	250 and 250.X	E11 and E11.X and E11.XX and E11.XXX
Other specified diabetes mellitus (for people aged <60 y)		E13 and E13.X and E13.XX and E13.XXX

Abbreviations: HBsAg, hepatitis B surface antigen; ICD, International Classification of Diseases.

^a^ Risk factors for hepatitis A that could not be sampled for through ICD 9/ICD 10 codes: occupational exposure to virus; intravenous drug use; travel in countries with high or intermediate endemic hepatitis A; close, personal contact with international adoptee; settings for exposure, including** **health care settings with services to injection or noninjection drug users or group homes and nonresidential day care facilities for developmentally disabled people.

^b^ Risk factors for hepatitis B that could not be sampled for through ICD9/ICD 10 codes: intravenous drug use; household contacts of HBsAg-positive people; residents and staff of facilities for developmentally disabled persons; health care and public safety personnel with reasonably anticipated risk for exposure to blood or blood-contaminated body fluids; incarcerated people; travel in countries with high or intermediate endemic hepatitis B.

^c^ Includes hemodialysis, peritoneal dialysis, home dialysis, and predialysis patients; people with diabetes mellitus aged younger than 60 years, shared clinical decision-making for people aged 60 years or older.
